# Peeking into a black box, the fairness and generalizability of a MIMIC-III benchmarking model

**DOI:** 10.1038/s41597-021-01110-7

**Published:** 2022-01-24

**Authors:** Eliane Röösli, Selen Bozkurt, Tina Hernandez-Boussard

**Affiliations:** 1 School of Life Sciences, Swiss Federal Institute of Technology (EPFL), Lausanne, Switzerland; 2 Department of Medicine (Biomedical Informatics), Stanford University, Stanford, CA USA; 3 Department of Biomedical Data Sciences, Stanford University, Stanford, CA USA

**Keywords:** Research data, Medical ethics

## Abstract

As artificial intelligence (AI) makes continuous progress to improve quality of care for some patients by leveraging ever increasing amounts of digital health data, others are left behind. Empirical evaluation studies are required to keep biased AI models from reinforcing systemic health disparities faced by minority populations through dangerous feedback loops. The aim of this study is to raise broad awareness of the pervasive challenges around bias and fairness in risk prediction models. We performed a case study on a MIMIC-trained benchmarking model using a broadly applicable fairness and generalizability assessment framework. While open-science benchmarks are crucial to overcome many study limitations today, this case study revealed a strong class imbalance problem as well as fairness concerns for Black and publicly insured ICU patients. Therefore, we advocate for the widespread use of comprehensive fairness and performance assessment frameworks to effectively monitor and validate benchmark pipelines built on open data resources.

## Introduction

The expansive availability of digital health data has led to a colossus of data-driven models to guide and improve healthcare delivery. This change of paradigm will and partially already does decisively shape medical diagnostics, drug discovery, clinical research and personalized medicine^1^. These tools approach and sometimes surpass expert clinicians in certain specialties^2^. Thereby, medical artificial intelligence (AI) has the potential to render healthcare more efficient and effective through better informed decisions and improved patient outcomes. However, emerging evidence suggests that many of these data-driven clinical decision support tools may be biased and not equally benefit all populations^3–6^. As AI makes progress to improve quality of care for some patients, others are left behind^7–10^. Particularly minorities and historically disadvantaged groups are at risk of suffering from unfair model predictions, as we have seen for example in the case of COVID-19^11,12^.

AI models are susceptible to bias since they learn themselves from biased data reflecting an intrinsically unjust healthcare system^13,14^. In the absence of tight controls, AI could hence unconsciously reinforce pre-existing biases through dangerous feedback loops^15,16^. To address these concerns, many recent studies, books and ventures have provided methodologies and frameworks for reporting breakdowns in model performance across different protected entities, for example gender, ethnicity or socioeconomic background. These approaches range from corporate Algorithmic Auditing and Consulting ventures to full ML life cycle bias screenings, fairness definition comparison studies or frameworks targeting a specific single protected entity^4,17–20^. While these efforts highlight pathways towards gaining a better understanding of the fairness and generalizability of data-driven models, the identification of a standard set of metrics for systematic, objective and comprehensive evaluation is still emerging. In particular, no universal notion of fairness exists as of yet. Rather, the inherent difficulties to mathematically capture and express the vague concept of fairness gave rise to a multiplicity of formal definitions, all of them valid in their own right^21,22^. However, most of these fairness definitions can be regrouped into three main classes as suggested by Corbett-Davis and Goel: anti-classification, classification parity and calibration^19^.

The risks of model bias and fairness concerns corrode the public’s trust and therefore critically hamper the successful adoption of clinical AI tools^23^. In addition to systematic fairness assessments, data sharing and transparency are key to build up trust, improve model quality and foster a better understanding of potential biases to enable effective mitigation. The Medical Information Mart for Intensive Care (MIMIC) is a prime example of such effort^24^. Its creation in 2011 constituted a paradigm change, being one of the first publicly available Electronic Health Record (EHR) databases. The resulting broad use has spurred the development of thousands of AI models. Furthermore, many communities view MIMIC as a gold standard for developing further EHR sandboxes to spur the development and testing of AI-driven research. MIMIC is also used for educational purposes – to train the next generation of AI developers. Therefore, understanding the inherent biases, the demographic representativeness and the risk of model overfitting in the MIMIC dataset is essential to guide these future endeavors.

Recently, Harutyunyan *et al*. built a MIMIC-III multi-task benchmarking pipeline focusing on four specific clinical prediction tasks: in-hospital mortality, physiological decompensation, length of stay and phenotype classification^25^. With the idea of an iterative improvement process in mind, this case study highlights the potential for validating and refining clinical decision support models developed in publicly accessible datasets. It focuses on the benchmark model of in-hospital mortality (IHM) since this constitutes one of the prime outcomes of interest in an ICU setting. Better understanding this benchmarking model is particularly relevant since it represents a typical use case for a publicly available EHR dataset and received great attention by the scientific community. In particular, several dozen other research teams have already used this benchmarking pipeline to assess different IHM modeling approaches with MIMIC-III data yet show a similar lack of comprehensive model evaluations^26–31^.

As studies like the publication by Harutyunyan *et al*.^25^ are forging new paths for benchmark models in open data, it is important to understand the implications of single-site, potentially biased benchmarks as well as the ubiquitous use of the MIMIC dataset to produce fair and equitable healthcare solutions. The aim of this study is to raise broad awareness of the pervasive challenges around bias and fairness in risk prediction models. To inform this discussion, we conducted an empirical case study to characterize the opportunities and limitations of the Harutyunyan benchmark model developed with MIMIC data. Specifically, our contribution is three-fold. First, we replicated the Harutyunyan study in the MIMIC database, including an evaluation of the cohort building process and the resulting demographic distributions. Second, we ran the Harutyunyan model in a separate EHR system from a different geographic location to test its generalizability. Third, we re-trained and tested the model in the independent validation set. At each stage, we applied a comprehensive fairness and generalizability assessment framework building on the fairness definition classification work from Sharad-Davis & Goel^19^ to characterize the risk of any undue bias towards certain demographic groups based on gender, ethnicity and insurance payer type as a socioeconomic proxy. Based on the insights gained from this work, we discuss the hurdles of developing fair algorithms using MIMIC data and highlight recommendations on how to cautiously monitor and validate benchmark pipelines arising from this important resource. In addition, understanding the limitations of models derived from MIMIC data can provide guidance to other initiatives to create further and even more powerful open-source EHR datasets.

## Results

### Case study framework

**Fig. 1:**
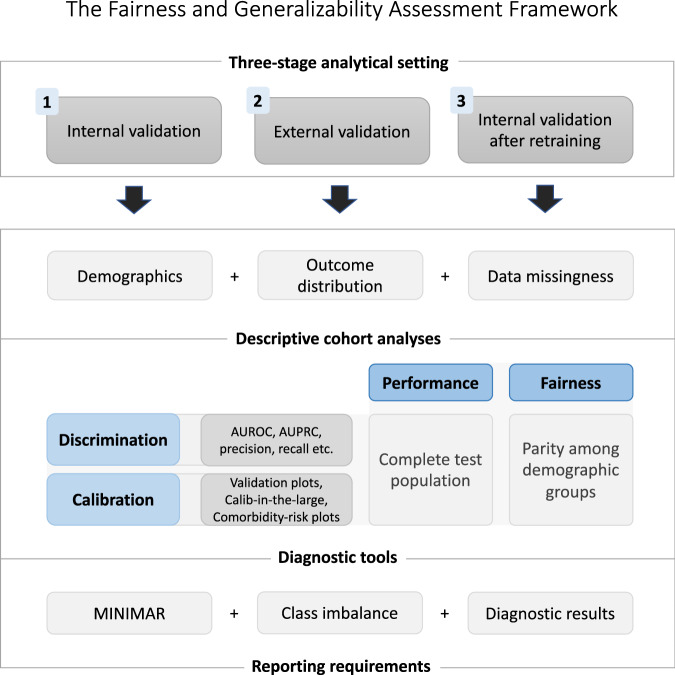
The fairness and generalizability assessment framework.

For greater clarity, the fairness and generalizability assessment framework used in this case study is first quickly presented here in Fig. 1 with more details available in the Methods section. The framework pulls from other existing methodologies and is based on a three-stage analytical setting: (1) internal model validation, followed by (2) external validation and (3) internal validation after retraining on the external data. At each stage, a fairness and generalizability assessment made up of three tasks is performed: (A) descriptive cohort analyses, (B) performance and fairness evaluations, and (C) comprehensive reporting. The cohort screenings should particularly focus on the cohort demographics, the outcome distribution and data missingness of the model variables. Many tools exist for performance and fairness evaluations, at the minimum a discrimination and calibration assessment should be done both on the test population level for performance analysis and on a demographic group level to check for parity of those metrics. Finally, comprehensive reporting guidelines should be followed, such as provided in MINIMAR^32^. Additionally, class imbalance should be discussed and all evaluation results of the model from task A and B should be provided.

### Case study

#### Descriptive cohort analyses

##### Cohort demographics

Table 1 shows the demographic distribution of patients, ICU stays and IHM rates across both datasets. STARR is one third the size of MIMIC and has a similar gender distribution. The age distribution is similar for young adults but STARR has fewer older patients above 70. The STARR dataset is more ethnically diverse with only roughly half of the patients being non-Hispanic White compared to two thirds in the MIMIC dataset. In terms of health insurance, roughly a third of MIMIC and STARR patients are covered by private insurance, and more than 50% is enrolled in Medicare. MIMIC’s overall IHM rate of 13.23% is almost a third higher than STARR’s rate of 10.18%. IHM increases with age, where STARR has consistently lower rates than MIMIC except for the youngest age group. Female patients have a higher IHM risk in both datasets, with the difference being more pronounced in STARR. Moreover, there is also a large range of variability for ethnic and socioeconomic groups.

**Table 1:** Characteristics of the MIMIC and STARR study cohorts.

	MIMIC			STARR		
	Patients n (%)	ICU stays n (%)	IHM rate (%)	Patients n (%)	ICU stays n (%)	IHM rate (%)
**Totals**	18’094	21’339	13.23	6’066	6’407	10.18
**Gender**						
Female	8’090 (44.7)	9’510 (45.0)	13.5	2’485 (41.0)	2’641 (41.2)	11.6
Male	10’004 (55.3)	11’629 (55.0)	13	3’581 (59.0)	3’766 (58.8)	9.2
**Age**						
0–17	0 (0.0)	0 (0.0)	0	0 (0.0)	0 (0.0)	0
18–29	782 (4.3)	873 (4.1)	5.6	275 (4.5)	291 (4.5)	7.2
30–49	2’680 (14.8)	3’171 (15.0)	9.3	879 (14.5)	958 (15.0)	8.8
50–69	6’636 (36.7)	7’921 (37.5)	11.1	2’660 (43.9)	2’814 (43.9)	9.1
70–89	7’043 (38.9)	8’065 (38.2)	16.5	2’076 (34.2)	2’161 (33.7)	11.7
90+	953 (5.3)	1’109 (5.3)	21.8	176 (2.9)	183 (2.9)	20.8
**Ethnicity**						
Asian	437 (2.4)	492 (2.3)	13.8	837 (13.8)	883 (13.8)	11.9
Black	1’480 (8.2)	2’016 (9.5)	9.2	329 (5.4)	354 (5.5)	9.3
Hispanic	568 (3.1)	679 (3.2)	8.1	945 (15.6)	1’015 (15.8)	11.4
White	12’851 (71.0)	15’043 (71.2)	12.9	3’199 (52.7)	3’361 (52.5)	8.7
Other	2’758 (15.2)	2’909 (13.8)	18.7	756 (12.5)	794 (12.4)	13.5
**Insurance**						
Medicare	10’337 (57.1)	12’286 (58.1)	15.3	3’144 (51.8)	3’321 (51.8)	10.5
Medicaid	1’489 (8.2)	1’813 (8.6)	10.3	944 (15.6)	1’006 (15.7)	10.3
Private	5’601 (31.0)	6’326 (30.0)	10.2	1’711 (28.2)	1’800 (28.1)	9.1
Other	667 (3.7)	714 (3.4)	11.6	267 (4.4)	280 (4.4)	12.1

Several ICU stays may be collected for a single patient.

##### Data missingness

Table 2 gives a diagnostic overview of the average amount of data available for each variable in each ICU stay. In MIMIC, capillary refill rate (CRR), fraction of inspired oxygen (FiO2) and patient height are missing in over two thirds of ICU stays (captured by the *None* column). CRR is missing altogether in 49 out of 50 patients and not available in STARR. On the other hand, there are 11 out of the 17 variables in MIMIC with less than 2% of the ICU stays having no measurements during the first 48 hours. STARR has better data coverage for FiO2 and height but generally suffers from higher rates of stays with completely missing data for a given variable. STARR also has four variables with full data coverage for more than 50% of the ICU stays in the cohort, whereas this indicator is below 20% for all variables in MIMIC. Finally, differences can also be seen when looking at the average number of data points retained per variable in the *Average* column across both datasets.

**Table 2:** Data coverage statistics for physiological model variables.

	MIMIC			STARR		
	None (%)	Full (%)	Average n(%)	None (%)	Full (%)	Average n(%)
Capillary refill rate	98.1	0	0.2 (0.3)	100	0	0.0 (0.0)
Diastolic blood pressure	1.2	14	43.4 (6.2)	11	8.9	20.2 (42.0)
Fraction inspired oxygen	70.5	0	3.0 (6.2)	43.5	0	3.5 (7.2)
Glascow coma scale						
Eye opening	0.9	0.1	14.8 (30.9)	14.3	0	6.7 (14.1)
Motor response	0.9	0.1	14.8 (30.8)	14.2	0	7.2 (15.1)
Total	41.8	0.1	8.8 (18.4)	14	0	9.8 (20.4)
Verbal response	1	0.1	14.8 (30.8)	14.7	0	5.6 (11.7)
Glucose	0.1	0	12.5 (26.0)	9.8	0.5	15.2 (31.6)
Heart rate	1.2	19.8	44.4 (92.4)	1.8	74.9	45.8 (95.4)
Height	81	0	0.2 (0.4)	9.7	76.8	42.9 (89.4)
Mean arterial pressure	1.2	13.3	43.2 (90.0)	11	8.9	20.2 (42.0)
Oxygen saturation	0.7	14.1	42.8 (89.3)	1.7	65.2	45.5 (94.9)
Respiratory rate	1.3	18.6	43.7 (91.0)	13.7	36.7	38.1 (79.3)
Systolic blood pressure	1.2	14.1	43.4 (90.4)	11	8.9	20.2 (42.0)
Temperature	2	0.4	15.7 (32.6)	3.8	1.3	20.0 (41.7)
Weight	27	0	1.5 (3.1)	4.8	81.1	45.3 (94.3)
pH	17.3	0	6.3 (13.1)	22.2	0	7.8 (16.3)

*None* relates to the percentage of ICU stays that do not have any data points for the given variable during the study period (first 48 h after ICU admission). *Full* captures the percentage of stays with available data for every hour during the 48 h analysis period. *Average* reports the mean number of data points usable out of a maximum of 48.

#### Performance evaluation

##### Model discrimination

The results for discriminatory power in all three framework stages are presented in Table 3.

**Table 3:** Evaluation metrics reported by the benchmark study^25^ and the three framework stages.

Metrics	MIMIC-trained model			STARR-trained model
	Benchmark study	(1) Internal validation	(2) External validation	(3) Internal validation
**Test data IHM rate**	—	11.56%	10.18%	10.19%
**AUROC**	0.862 (0.844, 0.881)	0.861 (0.842, 0.879)	0.827 (0.810, 0.843)	0.872 (0.839, 0.904)
**AUPRC**	0.515 (0.464, 0.568)	0.499 (0.452, 0.546)	0.408 (0.372, 0.446)	0.500 (0.403, 0.601)
**Accuracy**	—	0.896 (0.889, 0.903)	0.907 (0.903, 0.911)	0.912 (0.902, 0.921)
**Precision event**	—	0.618 (0.546, 0.692)	0.658 (0.591, 0.724)	0.783 (0.609, 0.944)
**Precision non-event**	—	0.910 (0.905, 0.914)	0.915 (0.912, 0.918)	0.915 (0.908, 0.923)
**Recall event**	—	0.255 (0.211, 0.299)	0.186 (0.156, 0.216)	0.184 (0.112, 0.265)
**Recall non-event**	—	0.979 (0.974, 0.984)	0.989 (0.986, 0.992)	0.994 (0.988, 0.999)

**Stage 1**. The test data is imbalanced with an IHM rate of 11.56%. Accounting for some variability due to random factors, the results for AUROC and AUPRC reported in the benchmark study could be reproduced. AUROC and accuracy are both well above 0.8, whereas AUPRC is lower. Precision and especially recall of the event instances are also lower as compared to non-events.

**Stage 2**. Testing the same model on STARR data with an IHM rate of 10.18%, AUPRC dropped by almost 0.1 points as compared to internal validation in stage one, whereas AUROC remained stable. Accuracy, precision for both outcomes and recall of non-events slightly increased, though these changes are mostly not significant at the 95% significance level. The event recall, however, falls below the 20% mark, implying that the model now only identifies one in five high-risk patients as such, compared to the one-in-four ratio achieved in internal validation.

**Stage 3**. Retraining the model on STARR data resulted in a visible improvement of AUPRC and AUROC as compared to the second stage results. However, the confidence intervals of the metrics became larger because the STARR test set is now much smaller. There is a noteworthy improvement in event precision but the recall of those instances remains extremely low.

##### Model calibration

**Fig. 2:**
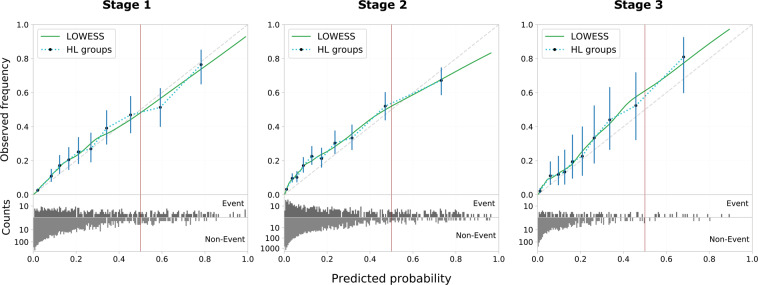
Validation plots of the three framework stages. Model calibration is shown alongside the distribution of risk predictions on the bottom (counts in log-scale). Calibration is assessed on ten Hosmer-Lemeshow risk groups based on exponential quantiles including 95% binomial proportion confidence intervals using Wilson’s score. In addition, the non-parametric smoothing line given by LOWESS is added. Ideal calibration is indicated by the diagonal dashed line in gray. The vertical red line indicates the decision threshold to give binary predictions on in-hospital mortality based on a risk percentage.

Figure 2 shows the validation graphs for all three framework stages.

**Stage 1**. The MIMIC-trained model appears well calibrated, with only slight risk underestimation in the low risk strata and risk overestimation for the high risk strata.

**Stage 2**. When testing the MIMIC-trained model on STARR data, its predictions remain reasonably well calibrated. However, risk underestimation for lower risk strata got more severe and is statistically significant.

**Stage 3**. The STARR-trained model shows consistent risk underestimation across all risk strata. Compared to the MIMIC-trained model predictions on STARR, risk underestimation decreased slightly for lower risk strata but is much worse for higher risk patients.

#### Fairness evaluation

##### Classification parity

**Fig. 3:**
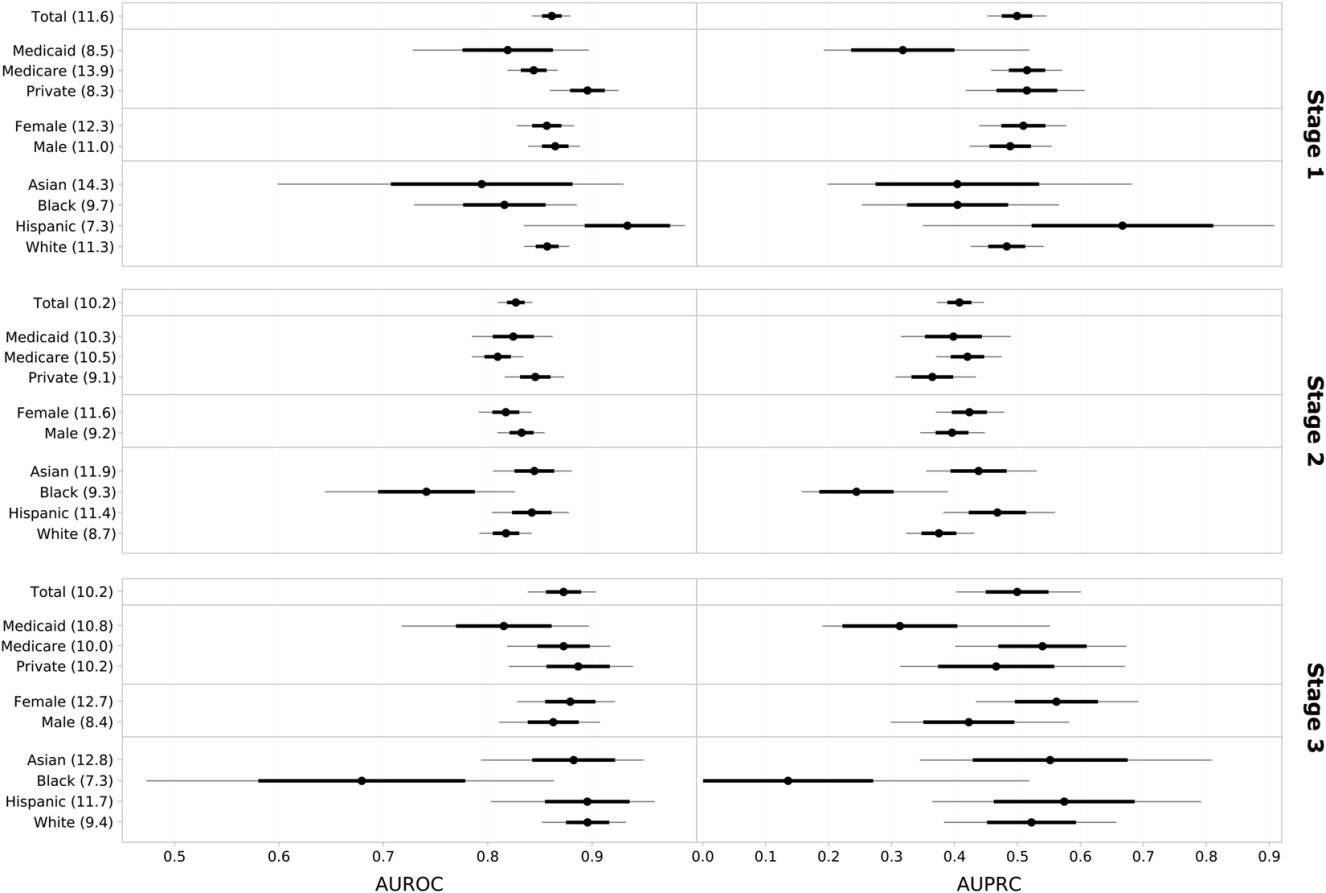
Classification parity plots of AUROC and AUPRC for the three analytical framework stages. In-hospital mortality rates (%) for demographic groups are added in parentheses after the group labels. 95% confidence intervals are illustrated by thin gray lines, standard deviations by bold black lines and median values by black dots.

Figure 3 shows the classification parity plots for the three stages of the case study framework. The plots need to be interpreted in the context of class imbalance as explained in the corresponding Methods section.

**Stage 1**. Two major points can be made here: Since patients with private insurance and Medicaid have very similar event rates (8.3% and 8.5%, respectively) but Medicaid patients get distinctively worse predictions as measured by both AUROC and AUPRC, classification parity is violated. A similar argument can be made for two ethnic groups, where non-Hispanic White patients have a higher IHM rate (11.3%) as compared to Black patients (9.7%) – which should disadvantage them – yet still achieve better scores for both discrimination measures. There are no notable performance differences for gender and the high model performance on Hispanic patients is linked to a much lower IHM rate than any other group (7.3%).

**Stage 2**. The external validation results of the MIMIC-trained model show no meaningful observable differences in performance for the different socioeconomic groups. The one percentage point lower IHM event rate for privately insured patients explains why their AUROC is slightly higher than for public insurance but falls behind for AUPRC. Medicaid patients receive much more accurate predictions in STARR than was the case for MIMIC. There are again no notable differences in terms of gender but stark disparities can be found for non-Hispanic White and Black patients: They have very similar IHM rates in STARR (8.7% and 9.3%, respectively), yet Black patients suffer again from comparably very bad model predictions. The performance on Asian and Hispanic patients is very similar and even slightly better than for White patients.

**Stage 3**. One can again note the larger confidence intervals due to a smaller test set in the last framework stage. Despite very similar event rates across the different socioeconomic groups, Medicaid once more suffers from noticeably worse predictions both in terms of AUROC and AUPRC. Also, female patients now tend to have more accurate predictions than male patients despite their higher event rate. Finally, Black patients now suffer from even stronger model discrimination with much worse prediction performance than all other ethnic groups. This result is reinforced by the event rate of 7.3% for Black patients in the test data, which is the lowest for any ethnic group and should support good model performance given the problem of class imbalance. Notably, the previous performance gap of non-Hispanic White patients as compared to Hispanic and Asian patients practically vanished after model retraining.

##### Calibration

**Fig. 4:**
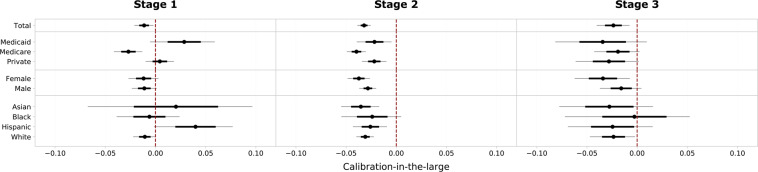
Plots of calibration-in-the-large under demographic stratification for the three analytical framework stages. The deviations of the predicted average risk from the observed average risk are shown. 95% confidence intervals are illustrated by thin gray lines, standard deviations by bold black lines and median values by black dots. The dashed line in red indicates optimal agreement between predicted and observed risk.

***Calibration-in-the-large.*** Figure 4 shows the calibration-in-the-large plots under demographic stratification for the three analytical framework stages.

**Stage 1**. The MIMIC-trained model indicates slight internal decalibration as the actual IHM risk is underestimated on average (10.4% predicted vs. 11.6% actual risk) despite being trained on data with an event rate of 13.5%. Risk calibration varies for socioeconomic groups: Private patients receive the best overall calibrated risk predictions, whereas the risk of Medicare patients is significantly underestimated and the risk of Medicaid patients slightly overestimated. Calibration remains stable for gender-specific groups but varies by ethnicity. While non-Hispanic White patients have the same calibration as the overall model, confidence intervals are longer for the ethnic minority groups Asian, Black and Hispanic and particularly the risk for Hispanic patients is overestimated.

**Stage 2**. Risk underestimation gets stronger when tested on STARR data: the MIMIC-trained model predicts a 7.0% risk given an actual event rate of 10.2% in the test data. Medicaid and privately insured patients are still comparably well calibrated, whereas the risk for Medicare patients is strongly underestimated. Also, the risk of female patients is now more strongly underestimated than it is for male patients and the differences in calibration for socioeconomic groups essentially disappear.

**Stage 3**. Retraining slightly improves decalibration but the model still consistently underestimates the IHM risk for all demographic groups. Female patients still have worse risk underestimation and Black patients have the best calibration-in-the-large despite extremely low discrimination performance.

**Fig. 5:**
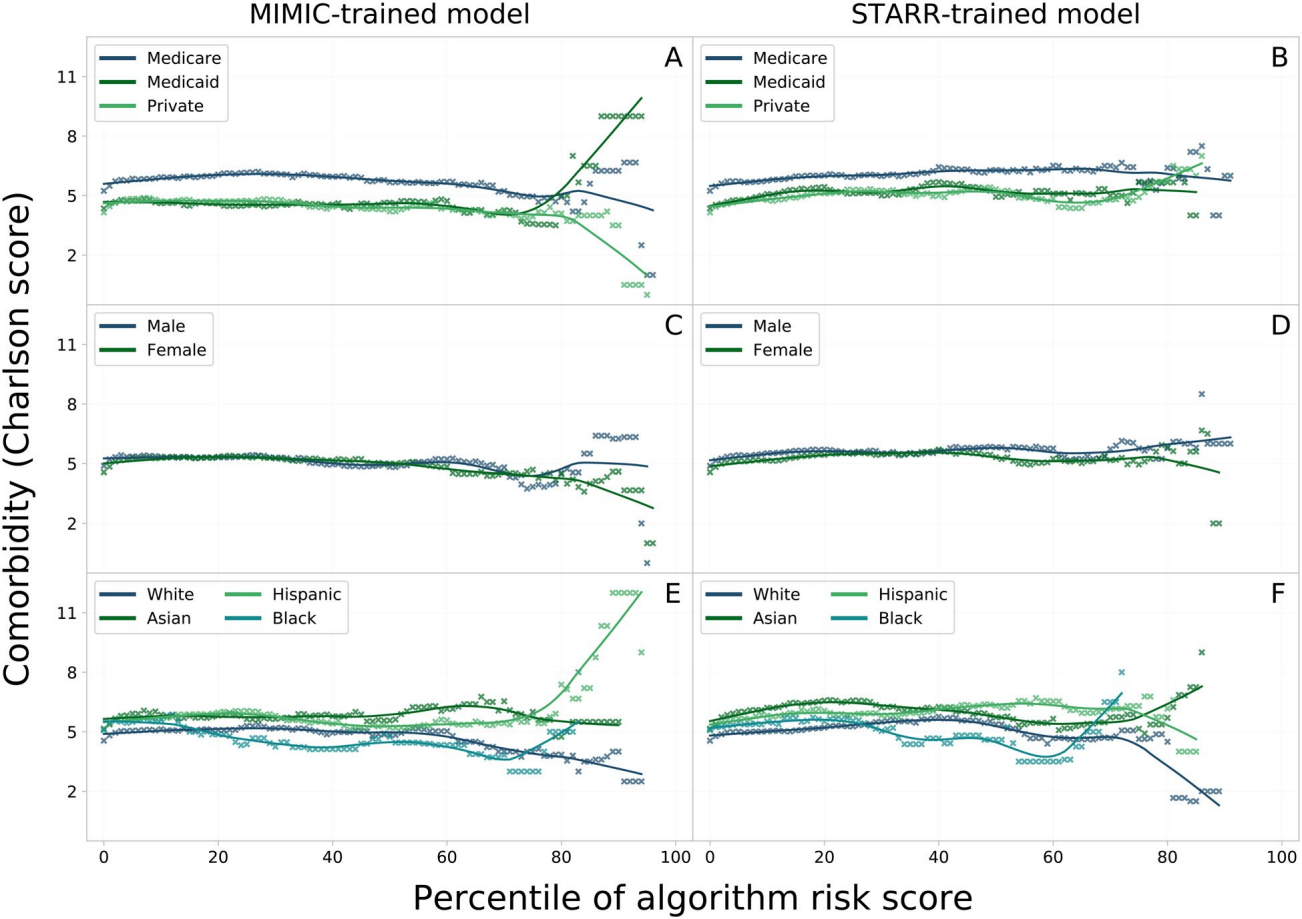
The percentiles of algorithmic IHM risk scores are plotted against patients’ comorbidities, quantified by the Charlson score. The curves are plotted for all demographic groups based on predictions on STARR data by the MIMIC and STARR-trained model. Plot A and B show groups by insurance payer type, plot C and D by gender, and plot E and F show the ethnic groups.

***Comorbidity-risk plots.*** The plots in Fig. 5 show the relation between algorithmic risk and comorbidity. Patient comorbidity is quantified by the Charlson score based on a weighted sum of the severity of 12 comorbidity factors that were shown to be indicative of the overall patient state of health. Overall, the plots do not clearly show a uniform trend of expected positive correlation. Rather, the curves stay roughly stable until the 70th algorithmic risk score percentile before starting to diverge in most demographic groups.

**Insurance type**. High-risk Medicaid patients are more likely to suffer from a heavy burden of comorbidities as shown by the spike in plot A whereas the converse holds true for high-risk patients with private insurance. That disparate trend can, however, not be observed in the risk predictions by the STARR-trained model (plot B). Finally, Medicare patients have a very stable relationship between algorithmic risk score and comorbidity. However, they visibly suffer from more comorbidities (about 1 index point) for a given risk score in both the MIMIC and STARR-trained model.

**Gender**. In the plots C and D, the curves show no significant differences in comorbidity burden given a low-to-medium algorithmic risk score percentile. The curves slightly diverge in both models for higher risk strata with male patients suffering from slightly more comorbidities.

**Ethnicity**. Generally speaking, Hispanic and Asian patients have a higher comorbidity burden than non-Hispanic White and Black patients for a given risk score as shown in plots E and F. Where non-Hispanic White and Asian patients of high-risk strata show a slight decline in the Charlson score in plot E, Black and particularly Hispanic patients experience a steep increase similar to Medicaid patients in plot A. The same, but even more distinct, pattern can be seen in plot F for non-Hispanic White and Black patients, whereas the trend reverses for Hispanic and Asian patients.

## Discussion

The MIMIC dataset provides a wealth of opportunities to develop, train, and educate; as a prime example, Harutyunyan *et al*.^25^ developed a benchmark study to predict IHM risk that has been replicated in multiple settings^26–31^. Given the magnitude of impact of this work, we performed an empirical evaluation of this model – a task generally done far too inconsistently and sparingly^33^. In this endeavor, we appreciated the authors’ efforts to facilitate model comparison through the development of easily reproducible benchmark models as well as their detailed explanations and general user-friendliness of their open-source code. While there are many strengths to this publication, we also identified several limitations related to class imbalance and fairness that require mitigation and transparent reporting. Specifically, we found three main problems to be addressed. First, the cohort and performance screening unmasked a typical class imbalance problem, where the model struggles to correctly classify minority class instances as demonstrated through low recall. Only every fourth to fifth high-risk patient is identified as such by the AI tool. Second, while the assessment showed the model’s capacity to generalize, the classification parity assessment revealed that model fairness is not guaranteed for certain ethnic and socioeconomic minority groups, but gender is unaffected. Finally, the calibration fairness study pointed to differences in patient comorbidity burden for identical model risk predictions across socioeconomic groups. These results highlight the extent of masked bias in high-quality scientific work and the need for thorough fairness evaluations. In light of the possible repercussions and the pervasiveness of bias in AI models, we provide a detailed analysis of the case study results and highlight recommendations on how to cautiously monitor and validate benchmark pipelines.

The empirical analysis of the Harutyunyan model suggests that class imbalance has a significant effect on this model’s performance in all three analytical framework stages. In the context of AI-guided clinical decision support, such limitations may threaten the model’s usability. As seen in particular from this model, recall rates as low as 25% or less should raise important concerns regardless of the model’s potential future application fields and use cases. In fact, this specific model’s endpoint may not be clinically relevant in many settings, but the pandemic has proven that there are moments where doctors increasingly look for tools to help them triage patients in a resource-scarce or momentarily overstrained setting. In a slightly different context, a case in point of such a tool is the Care Assessment Need (CAN) score deployed for almost a decade by the Veterans Health Administration in the US. It calculates weekly CAN scores for all Veterans who receive primary care services within the VA, including 90-days and 1-year mortality endpoints^34^. This tool has also been shown to be amenable to COVID-19 mortality risk stratification repurposing to support clinical decision making in a system under duress^35^.

One of the biggest dangers of model bias and class imbalance, such as exhibited by this benchmark model, is the fact that such intrinsic modeling problems frequently get hidden by inadequate and too simplistic summary performance metrics such as accuracy, or to a lesser degree even in current reporting standards such as AUROC^36–38^. The accuracy paradox explains how this pitfall may cause misleading conclusions in the case of unbalanced datasets by failing to provide comprehensible information about the model’s discriminatory capabilities^39^. Various targeted data and algorithm-level methods have been developed to effectively mitigate the inherently adverse effects of class imbalance^40^. But even if such mitigation methods may, in some cases, decisively help in alleviating the repercussions of data imbalance on performance, there is still a critical need to address the remaining negative impact. Given the still widespread neglect surrounding the proper handling of class imbalance and the associated far-reaching negative consequences^37^, it is urgent to finally work towards broad adoption of adequate performance evaluation procedures and reporting standards, such as provided in MINIMAR^32^, taking into account the characteristic challenges arising from class imbalance. More specifically, we advocate for the cessation of simplistic performance reporting solely based on accuracy or AUROC since performance metric choices have far-reaching implications on the study’s conclusions, particularly in the presence of data imbalance. Therefore, it is imperative that scientific journals now go beyond current reporting guidelines and start broadly requiring publications to explicitly state the class (im)balance and, in the case of pronounced skew, to require the reporting of further metrics beyond accuracy and AUROC.

Despite the inherent difficulties associated with mathematical definitions of fairness^19,21^ and the analytic limitations imposed by class imbalance^37^, significant differences for both examined fairness criteria were found. The classification parity assessment revealed important performance differences for socioeconomic and ethnic groups, but not with respect to gender. Medicaid patients receive significantly worse predictions compared to patients with private insurance in the internal validation assessments of both the MIMIC and STARR-trained model (stage one and three). Studying ethnic disparities, Black patients suffer from significantly lower model prediction performance as compared to non-Hispanic White patients in all assessment stages. Part of this bias might stem from the low representation of Black patients in both datasets, yet the two other ethnic minority groups, Asians and Hispanics, did not show such pronounced bias. Evidently, more research is warranted to better understand the underlying causes of these observed differences.

Complementary to classification parity, the fairness calibration analysis revealed further fairness disparities for publicly insured patients. Particularly Medicare patients consistently suffer from the strongest decalibration as their risks are severely underestimated. Since the Medicare population tends to be distinctively older by the nature of its inclusion criteria resulting in an elevated IHM rate, these results are however not surprising in the context of the class imbalance problem. Studying the relationship of comorbidity burden and algorithmic risk predictions paints a similar picture as Medicare patients consistently have more comorbidities for any risk score percentile. In the hypothetical case of deploying such a model in an ICU setting, older patients would potentially be at risk of not receiving the appropriate care resources they would need to improve their chances of survival.

Another interesting phenomenon in the study of comorbidity-risk associations are the randomly seeming spikes for higher risk percentiles in many demographic groups. They may be partially explained by random fluctuations due to the quickly decreasing sample size as the algorithmic risk score increases. Another explanation, however, might be that the IHM risk of those high-risk strata patients has strongly varying dependencies according to the characteristics of the demographic group. The opposing spiking behavior of Medicaid and privately insured high-risk patients is particularly interesting. The steep positive association of risk score and comorbidity burden in Medicaid patients suggests that their predicted likelihood of death in or after an ICU stay is disproportionally driven by comorbidities. On the other hand, patients with private insurance seem much more likely to receive such a high model risk score because of a sudden unexpected event unrelated to their comorbidities. This disparity in comorbidity burden points to a more systemic problem in our healthcare system, where socioeconomically disadvantaged patients receive unequal quality of care, contributing to many preventable deaths^13,14,41,42^.

Bias in a deployed AI-guided clinical decision support may have long-lasting and severe negative consequences for the affected patient groups. In this specific case, particularly Black patients and patients under public insurance would be at risk, though other minority groups may also suffer from underrepresentation^3–6^. These results build on Obermeyer’s previous work and strengthen the case that Black patients and other systemically disadvantaged groups are the ones left behind by AI revolutionizing standards of care^9^. Not identifying and addressing such hidden biases would lead to their propagation under the mantle of AI, thereby further exacerbating the health disparities faced by minority populations already today^7–10^. Moreover, such wide-ranging repercussions would also destroy public trust and hinder the further adoption of AI tools capable of decisively improving patient outcomes^21^.

While this case study is focused on the evaluation of the risk-prediction behavior of a MIMIC-trained model, it is important to consider the underlying data and its fit for purpose. Much of our historical healthcare data include inherent biases from decades of a discriminatory healthcare system^13,14,43^. For example, several studies have documented that non-White patients were less likely to receive an analgesic for acute pain, particularly when the underlying reason is difficult to quantify, such as a migraine or back pain^44,45^. This disparity becomes embedded in the data and therefore a model learning from these data can only regurgitate the biases in the data itself. This highlights the need for diverse stakeholder involvement throughout the design, development, evaluation, validation and deployment of an AI model to understand how and where these biases may occur in the data and potential mitigation strategies, such as discussion with a nurse/clinician about different levels of missingness for a particular variable^46^. Ultimately, solutions ready for safe deployment must be developed through a joint team effort involving people on the ground, knowledge experts, decision makers, and end-users.

Our work has far-reaching implications. First, it sheds light on the negative repercussions of disregarding class performance disparities in the context of skewed data distributions – a challenge still largely neglected but impacting many areas of AI research and requiring systemic changes in model evaluation practices and reporting standards^36–38^. Secondly, it showcases the importance of thorough external model validation prior to its use as a benchmarking tool. And finally, models should also systematically undergo fairness assessments to break the vicious cycle of inadvertently perpetuating the systemic biases found in society and healthcare under the mantle of AI. Particularly, the modeling of certain research questions on single-centered datasets like MIMIC is at an elevated risk of embedded systemic bias and the widespread use of only a handful of large public datasets further escalates the negative consequences thereof. Our work shows that particularly Black and socioeconomically vulnerable patients are at higher risk of inaccurate predictions by the model under study. These findings are of particular importance since this model has already been used several dozen times to benchmark new modeling techniques without any prior performance or fairness checks reported^26–31^.

This study has limitations. First, the fairness assessment is significantly limited by the model’s underlying problem of class imbalance. However, the results obtained under these unmodifiable constraints are statistically sound and meaningful. A second limitation is the lack of multi-center data, which would further strengthen the generalizability of the study findings, although two academic settings were evaluated in this study. 10.25740/tb877wd0973). Finally, the study is also limited by the similarity of the two datasets since STARR originates, like MIMIC, from an affluent academic teaching hospital setting. Yet the large geographic distance resulting in a different demographic patient population mix provides a good basis for a first model generalizability assessment.

In the present era of big data, the use of AI in healthcare continues to expand. An important aspect of the safe and equitable dissemination of AI-based clinical decision support is the thorough evaluation of the model and its downstream effects, a step that goes beyond predictive model performance to further encompass bias and fairness evaluations. Correspondingly, regulatory agencies seek to further initiate and develop real-world performance requirements and test beds, using MIMIC as a gold standard^10^. A crucial step to achieving these goals is open science. Yet as MIMIC is often viewed as an exemplar of open science, understanding its limitations, through for example case studies, is an imperative. In our evaluation of an IHM benchmarking model, we found challenges around class imbalance and worse predictions for Black and publicly insured patients. Going further, we also saw that model performance measured by AUROC was the center of evaluation and other important assessments, such as minority class performance, risk of bias and concerns around model fairness, were missing or not adequately reported. The repercussions from such non-comprehensive evaluation frameworks are a safety concern of entire populations, where the most vulnerable will ultimately suffer the most. Hence, this study cautions against the imprudent use of benchmark models lacking fairness assessments and external validation in order to make true progress and build trust in the community.

## Methods

### Data sources

#### MIMIC

MIMIC-III (v1.4), short for Medical Information Mart for Intensive Care, is an extensive single-centered database comprising EHRs of patients admitted to an intensive care unit (ICU) at the Beth Israel Deaconess Medical Center, an academic teaching hospital of the Harvard Medical School in Boston US^24^. The ICU data is deidentified and spans over a decade between 2001 and 2012. The most recent version 1.4 at the time of study is used here.

#### STARR

The STAnford medicine Research data Repository (STARR) contains electronic health record data from Stanford Health Care, the Stanford Children’s Hospital and various other ancillary clinical systems^47^. The data used for this study spans ICU stays from November 2014 to July 2019.

#### Demographic factors

Gender, insurance type and ethnicity are the three main demographic attributes considered in this case study. Gender is encoded in a binary fashion as either female or male as there are no other reported categories in both datasets. There are no missing values for any ICU stay in both datasets.

Health insurance type is included as a socioeconomic proxy. There are three major insurance types available in the United States: The public programs Medicare and Medicaid, and private insurance providers. Medicare is a federal social insurance program and anyone over 65 years or with certain disabilities qualifies for it, whereas Medicaid provides health insurance to very low-income children and their families. Insurance data was therefore mapped to the four distinct categories Medicare, Medicaid, private and other insurance. Neither MIMIC nor STARR has missing data for insurance type.

Following the terminology used in the MIMIC database, the term ethnicity is used here in the following, summarizing fashion: Patients self-reporting as of White race and non-Hispanic ethnicity are coded as *White*, Asian and Black patients are, independently of their Hispanic background, coded as *Asian* and *Black* respectively, and all other patients of Hispanic origin are coded as *Hispanic*. Patients of other races such as American Indian and Pacific Islander are regrouped under the catch-all option *Other*. Hence, patients were mapped in both datasets onto a total of five mutually exclusive and collectively exhaustive groups.

### Benchmark cohorts

#### MIMIC

The MIMIC benchmark cohort was reconstructed based on the publicly available source code of the benchmark model^25^. There are two major steps in the data processing pipeline. First, the root cohort is extracted based on the following exclusion criteria: Hospital admissions with multiple ICU stays or ICU transfers are excluded to reduce any possible ambiguity between outcomes and ICU stays. Moreover, patients younger than 18 are excluded as well. Finally, event entries are only retained if they can be assigned to a hospital and ICU admission and are part of the list of 17 physiological variables used for modeling (cf. Table 2). From the root cohort, the IHM cohort can be extracted by filtering for ICU stays that have a known length-of-stay of more than 48 hours and contain observation data during that initial time window. For a more detailed description of the cohort building process, the reader is referred to Harutyunyan’s publication^25^.

#### STARR

The processing flow of STARR data is very similar to MIMIC but has been adapted and optimized for its characteristics. Most importantly, we use identical exclusion criteria and data processing steps that were applied for the MIMIC cohort. More detailed information can be found on our GitHub repository

### Benchmark model

Mortality risk prediction is generally formulated as a binary classification problem using observational data from a limited time window of typically 12–24 hours following hospital admission. Harutyunyan’s model uses a longer 48-hour window inspired by the PhysioNet/CinC Challenge 2012 to facilitate capturing any change in patterns affecting patient acuity^25^. For in-hospital mortality, the target label captures whether a given patient has died before being discharged. The channel-wise long short-term memory (LSTM) model without deep supervision has been selected as the study focus based on its superior reported AUROC performance among the five developed non-multitask models by Harutyunyan *et al*.^25^. The selected benchmark model is a modified version of the standard LSTM where all variables are first independently pre-processed with an individual bidirectional LSTM layer instead of working directly on the full matrix of clinical events as usual. More details about the LSTM model architecture, the data preprocessing and the parameters of the training procedure can be found in Harutyunyan’s publication^25^.

### Three-stage analytical framework

The case study consists of three stages – descriptive cohort analyses, and performance and fairness evaluations – implemented across a three-stage analytical framework: (1) internal model validation on MIMIC data, followed by (2) external validation and (3) internal validation after retraining on STARR data.

#### Descriptive cohort analyses

After data selection and processing, the demographics of the resulting cohorts are analyzed by looking at the distributions of patient characteristics and the outcome in terms of gender, age, ethnicity and insurance type (cf. Table 1). Moreover, data missingness is quantified by looking at three main factors for each physiological variable: The percentage of ICU stays with (1) completely missing data and (2) fully available data during the 48 h observation window, as well as (3) the mean number of data points retained out of a maximum of 48, with the percentage in parentheses (cf. Table 2).

#### Performance evaluations

Prediction models should both be well calibrated and have high discriminatory power. The specific application context determines the relative importance of these two objectives.

##### Model discrimination

This study uses the threshold metrics precision, recall and accuracy with a risk threshold of 0.5 as implemented by Harutyunyan *et al*.^25^. In addition, two AUC metrics are used: (1) The area under receiver-operator curve (AUROC) and (2) the area under precision-recall curve (AUPRC). While random binary classifiers’ AUROC is always at 0.5, the baseline AUPRC is given by the event rate. This makes AUPRC especially useful and informative when dealing with moderately to highly skewed classes as precision is directly influenced by class imbalance^36^.

To create empirical 95% confidence intervals for the different discrimination metrics, bootstrapping has been used by independently resampling *K* times with replacement from the original test dataset to mimic different test populations. In accordance with Harutyunyan’s analysis protocol, *K* was set to 10’000 in this study^25^.

##### Model calibration

The Hosmer-Lemeshow (HL) goodness-of-fit test is a common tool to study calibration for binary risk prediction models. However, given its strong sensitivity to the chosen number of risk groups and cutoffs significantly impacting the conclusions regarding model fit^48^, the focus is put on graphical illustrations of calibration instead. Validation graphs as proposed by Steyerberg *et al*. are used to examine overall model calibration^49^. It is an augmented version of a calibration plot, where the mean expected and observed risk score data pairs are plotted for each HL risk group against the diagonal line, which is indicative for optimal calibration. The grouping protocol for the HL risk groups is adapted to the specifics of this modeling problem. The number of classes is set to the default value ten but splitting by quantiles or percentiles could not be used due to strong class imbalance in the data. Therefore, a tailored splitting approach had to be developed, termed exponential quantiles. The formula $f\left(q\right)={q}^{1/5}$ adjusts the quantiles *q* to work with skewed data. The exponent should be individually adapted to the specific data skew. Three additional features are added to this calibration plot: (1) The risk prediction distribution on the bottom of the graph for both outcomes gives a visual impression of the discriminatory capabilities. (2) 95% risk group confidence intervals help in dealing with the problems associated with the grouping procedure. These binomial proportion confidence intervals have been computed based on Wilson’s score. (3) The LOWESS algorithm, short for Locally Weighted Scatterplot Smoothing, gives calibration information independent of the grouping scheme (smoother span = 0.5).

#### Fairness evaluations

Corbett-Davis and Goel identified three major classes of fairness definitions^19^: anti-classification, classification parity and calibration. In this work, we only study the latter two definitions since anti-classification, the requirement to exclude any protected patient attributes, is already respected in Harutyunyan’s model.

##### Classification parity

This fairness concept asks for equal predictive performance across any protected groups as measured by commonly used threshold and ranking-based metrics^19^. The specific choice of examined metrics is an important design choice and should be adapted to the problem setting. In this case study, classification parity is assessed on the metrics AUROC and AUPRC to account for both the need to have a high-performing model but being aware of the challenges resulting from class imbalance. In fact, assessing classification parity gets even more challenging when there is a combination of skewed cohort data and differing performance for the two classes like it is the case for the IHM model under study. In an attempt to alleviate or even eliminate the far-reaching constraints of class imbalance on the fairness assessment framework, we tested the use of the synthetic minority oversampling technique (SMOTE) in the model training procedure, but without success^50^. In such a situation, valid conclusions about model fairness can only be drawn if taking the varying event rates across demographic groups into account since they directly impact AUROC and AUPRC. AUPRC is better adapted than AUROC to deal with data skew but these values cannot be directly compared either since the baseline performances are determined by the event rates^51^. Observed performance differences can therefore only be attributed to unfair model predictions in two scenarios such that the effect of class imbalance can be excluded: (1) the demographic groups have similar event rates or (2) their event rates are the converse of what the metric would imply (i.e., higher event rate yet still higher performance contrary to the expected negative effect of class imbalance or vice-versa). Hence, classification parity results can only be interpreted in close context with the IHM rates indicated in parentheses after the group labels in Fig. 2.

##### Calibration

This fairness definition requires that outcomes should be independent of protected attributes conditional on risk estimates. Therefore, calibration is not supposed to be substantially different across demographic groups. Two different approaches, calibration-in-the-large and comorbidity-risk plots, are used in this study to examine calibration under demographic stratification. They are better adapted to smaller datasets than the validation plots used in the predictive model performance evaluation phase.

*Calibration-in-the-large*. This calibration assessment consists in comparing the average rates of predicted and observed outcomes. The calibration-in-the-large plots quantify the observed difference in average predicted and observed risk in each bootstrap sample and build confidence intervals based on the relative differences. Confidence intervals centered around zero as indicated by the red dashed line are indicative for good calibration. Thereby, it is possible to diagnose systematic risk over- or underestimations for certain demographic subpopulations but the plots do not give any information on local model calibration as non-uniform decalibrations can simply average out.

*Comorbidity-risk plots*. Extending the definition of calibration, a model can also be examined for systematic disparities across demographic groups in patient comorbidity conditional on risk score percentiles. Comorbidity provides a good representation of the patient’s overall health by mainly focusing on chronic diseases. In this study, the Charlson comorbidity index based on a weighted sum of the severity of 12 comorbidity factors with an observation window of two years prior to ICU admission has been used for the computational analysis^52^. Intuitively, higher comorbidity scores should correlate with higher predicted IHM risk – independently of demographic factors. This hypothesis is analyzed for risk predictions by both the MIMIC-trained and STARR-trained model on the full STARR cohort. The analysis is limited to STARR test data since MIMIC is a database containing exclusively information about ICU stays, and not complete patient EHRs, hence not allowing to compute accurate Charlson comorbidity scores on those patients as well.

## IRB Statement

This study was approved by Stanford University’s Institutional Review Board.

## Previous Presentations

The results of this study have not been previously presented.

## Data Availability

MIMIC-III data is available on the PhysioNet repository^53^ and made widely accessible to the international research community through a data use agreement. The STARR database contains fully identifiable patient data and can therefore not be shared with the public. Access to the STARR database is restricted to investigators identified on IRB protocols, which may be pursued through a formal collaboration with the corresponding author.

## Code availability

Harutyunyan’s code for its benchmark suite is available on a Zenodo repository^54^. Our code to reproduce the results and apply the analytical fairness and generalizability assessment framework also to other contexts is available on GitHub (10.25740/tb877wd0973)^55^.
